# Associations between Water, Sanitation and Hygiene (WASH) and trachoma clustering at aggregate spatial scales, Amhara, Ethiopia

**DOI:** 10.1186/s13071-019-3790-3

**Published:** 2019-11-14

**Authors:** Forest M. Altherr, Andrew W. Nute, Mulat Zerihun, Eshetu Sata, Aisha E. P. Stewart, Demelash Gessese, Berhanu Melak, Tigist Astale, Gedefaw Ayenew, E. Kelly Callahan, Melsew Chanyalew, Bizuayehu Gashaw, Lance A. Waller, Zerihun Tadesse, Scott D. Nash

**Affiliations:** 1 Los Alamos National Laboratory, Los Alamos, NM USA; 2 The Carter Center, Atlanta, GA USA; 3 The Carter Center, Bahir Dar, Ethiopia; 4 The Carter Center, Addis Ababa, Ethiopia; 5 Amhara National Regional State Health Bureau, Amhara, Ethiopia; 6 Emory University, Atlanta, GA USA

**Keywords:** *Chlamydia trachomatis*, Facial cleanliness, Spatial analysis, Trachoma

## Abstract

**Background:**

Trachoma is the leading infectious cause of blindness globally. The WHO has recommended the SAFE (Surgery, Antibiotics, Facial cleanliness and Environmental improvements) strategy to eliminate trachoma as a public health problem. The F and E arms of the strategy will likely be important for sustained disease reductions, yet more evidence is needed detailing relationships between hygiene, sanitation and trachoma in areas with differing endemicity. This study addressed whether the regional differences in water, sanitation, and hygiene (WASH) variables were associated with the spatial distribution of trachomatous inflammation-follicular (TF) among children aged 1 to 9 years in the Amhara National Regional State of Ethiopia.

**Methods:**

Data from 152 multi-stage cluster random trachoma surveys were used to understand the degree of clustering of trachoma on two spatial scales (district and village) in Amhara using a geographical information system and the Getis-Ord G_i_* (d) statistic for local clustering. Trained and certified graders examined children for the clinical signs of trachoma using the WHO simplified system. Socio-demographic, community, and geoclimatic factors thought to promote the clustering of the disease were included as covariates in a logistic regression model.

**Results:**

The mean district prevalence of TF among children aged 1 to 9 years in Amhara was 25.1% (standard deviation = 16.2%). The spatial distribution of TF was found to exhibit global spatial dependency with neighboring evaluation units at both district and village level. Specific clusters of high TF were identified at both the district and the village scale of analysis using weighted estimates of the prevalence of the disease. Increased prevalence of children without nasal and ocular discharge as well as increased prevalence of households with access to a water source within 30 minutes were statistically significantly negatively associated with clusters of high TF prevalence.

**Conclusions:**

Water access and facial cleanliness were important factors in the clustering of trachoma within this hyperendemic region. Intensified promotion of structural and behavioral interventions to increase WASH coverage may be necessary to eliminate trachoma as a public health problem in Amhara and perhaps other hyper-endemic settings.

## Background

Trachoma is caused by repeated ocular infections with the bacterium *Chlamydia trachomatis* and is the leading infectious cause of blindness globally [1, 2]. Since 1998, the World Health Organization (WHO) and the Global Alliance to Eliminate Blinding Trachoma by 2020 (GET 2020) have relied on a multifaceted strategy to progress towards elimination of trachoma as a public health problem. This strategy, known by the acronym SAFE, relies on *surgery* to correct trachomatous trichiasis, *antibiotics* to treat infection, the promotion of *facial cleanliness*, and *environmental improvements* to interrupt transmission and reduce the suitability of the habitat for the physical vector *Musca sorbens* [3, 4].

Amhara National Regional State, Ethiopia is home to approximately 20 million people and many administrative districts (known locally as woredas) are endemic for trachoma [5]. Based on baseline prevalence data, the SAFE strategy was scaled up throughout all of Amhara starting in 2007 [6]. Population-based trachoma impact surveys, conducted following approximately 3 to 5 years of SAFE, demonstrated a decrease in trachomatous inflammation-follicular (TF) among children aged 1 to 9 years [5, 7]. However, this was not the case in all districts [8]. Moreover, TF prevalence appeared to be spatially heterogeneous post-SAFE, with progress observed in some districts while others remained hyperendemic. This analysis used cross-sectional survey data to describe the spatial clustering of TF among children aged 1 to 9 years in Amhara at two important aggregate spatial scales, the district and the village (known locally as gotts). Further analysis aimed to identify the environmental, household and behavioral factors associated with a high burden of TF at each scale.

## Methods

### Survey methods

Multi-level cluster-random surveys were conducted with consistent methodology in all 152 districts of Amhara between 2011 and 2016. The survey methods used for sampling, grader training, electronic data capture, and many variable definitions have been described elsewhere [5, 8–11]. Briefly, within each district, the village was the primary sampling unit and a compact segment of approximately 30 to 40 households within the village was the secondary sampling unit [5, 12]. The cumulative number of previous rounds of Mass Drug Administration (MDA) of antibiotics within each district prior to its impact survey (as reported annually to the International Trachoma Initiative) was applied to each village surveyed within the applicable district (range: 3–11 rounds) [13]. The presence of a health care facility and the presence of a paved road in the village were directly observed by the survey team.

The surveys captured household data related to hygiene and sanitation by interviewing an adult household member, most often the head female, in Amharic. Definitions for household variables not discussed here are consistent with previously published trachoma impact survey data as well as the WHO Joint Monitoring Programme guidelines where applicable [8, 14]. Data collected pertaining to latrine use were not available for all surveys and were excluded from the analysis. A dichotomous variable represented the highest education any adult in the household had completed as any formal education or none. Household crowding was defined as greater than 5 persons per household regardless of the physical size of the household [15].

After interviewing the head of household, recorders enumerated all living household members along with their age and sex. Conjunctival examinations were then performed on present household members to estimate the prevalence of all 5 signs of trachoma based on the WHO simplified trachoma grading system [16]. During this examination process, facial cleanliness among children aged 1–9 years was directly observed by the grader and was defined as the absence of any nasal and ocular discharge on the observed child’s face.

### Data analysis

Survey weights were calculated using the inverse of the 2-stage joint sampling probability [8].

Estimates for prevalence were produced using svy commands in STATA version 14.0 (STATA Corporation, College Station TX, USA). Weighted estimates of district and village level TF prevalence among children aged 1 to 9 years were mapped using an Adindan projection for Universal Transverse Mercator zone 37 North in ArcMap version 10.4.1 (ESRI, Redlands, CA, USA). Village point location data was calculated in decimal degrees by averaging the latitude and longitude values of all participating households within the village. Likewise, for each district the geographic centroid was calculated based on the GPS locations of selected villages.

We performed a series of spatial analyses, beginning with global assessments of spatial autocorrelation and continuing to tests of local variation in the observed autocorrelation to assess potential spatial influence between neighboring evaluation units (districts or villages) [17]. A global Moran’s *I* (implemented *via* ArcMap) suggested an overall pattern of positive spatial autocorrelation in TF prevalence and identified the average spatial scale of clustering. Specifically, the global analysis suggested that spatial neighbors defined *via* a 25-km fixed distance band yielded the peak in overall spatial autocorrelation between villages (where the average nearest neighbor distance between villages was 4.84 km, standard deviation 3.26 km).

Next, utilizing GeoDa version 1.8 (Luc Anselin, Chicago, IL, USA), we defined connectivity maps at the district scale, where we found a neighborhood consisting of the 8 nearest neighbors best captured the observed spatial relationships. We defined a spatial weights matrix corresponding to this neighborhood definition and used this throughout the remainder of our analyses.

Moving from global to local estimates, we next used the Getis-Ord G_i_* (d) (*via* ArcMap) local statistic to identify high prevalence clusters (“hotspots”) among the 152 districts and 1558 villages in the analysis. At the two spatial scales, we calculated the Getis-Ord G_i_* (d) statistic using the survey-weighted prevalence estimates for each feature, and the spatial weights matrix for each spatial scale defined above. To reduce the potential for false positive results, we used a false discovery rate adjustment to account for multiple testing [17]. Hotspots were identified with 90% confidence for districts and 95% confidence for villages and coded as a new dichotomous variable that was used as the outcome in logistic regression models. The significance value (*P*-value) associated with the local Getis-Ord G_i_* (d) test statistics served as a measure of the unusualness of potential local hotspots of prevalence. The significance threshold for district hotspots was adjusted to 0.1 in order for the number of clusters to be large enough to perform a standard maximum likelihood based logistic regression. This allowed us to perform similar analysis on the districts and villages to enhance methodological comparison between spatial scales. All districts and villages identified with significantly low local prevalence (coldspots) were grouped into a non-hotspot category to serve as the comparator group.

For variables collected as part of the survey, the weighted estimates corresponding to the spatial scale being analyzed, district and village were used as the covariates of interest. To explore the potential impact of climate variables, we imported raster surfaces for average annual temperature, average annual precipitation, and altitude from BioClim Global Climate Datasets [18]. We summarized data for each district by extracting the average point values from that district, and the geographic centroid for each village served as the location for the village-level extracted geoclimatic variables.

To complete our analyses, two separate regression analyses were performed to assess the factors contributing to residence in a hotspot at the district and village levels. Variables were considered significant to incorporate in the model selection process if the *P*-value was < 0.05. Collinearity was assessed prior to model selection using a condition index greater than 30 and a variable decomposition factor greater than 0.5 as threshold values [19]. All water, sanitation, and hygiene (WASH) variables were included in the model until covariates unrelated to WASH were assessed for inclusion in the model. Manual backwards stepwise selection assessed the model’s performance after exclusion of different sets of independent variables from the model. Model fit was assessed with the Akaikeʼs information criterion (AIC) where the best-fit model was selected as exhibiting the lowest AIC. The final model fit was again examined with different combinations of the exposure variables to select a high-performance model. The final models were assessed for discriminatory performance using a receiver operator curve (ROC) and the final model’s fit to the data was examined using the Hosmer-Lemeshow test.

## Results

Between 2011 and 2016, field teams surveyed 1558 villages in all 152 districts of Amhara, enumerating 282,400 individuals of whom 202,312 (71.6%) were examined for clinical signs of trachoma. Among all individuals enumerated, 75,144 were children aged 1 to 9 years and 69,236 (92.1%) of these children were examined for clinical signs of trachoma.

**Fig. 1:**
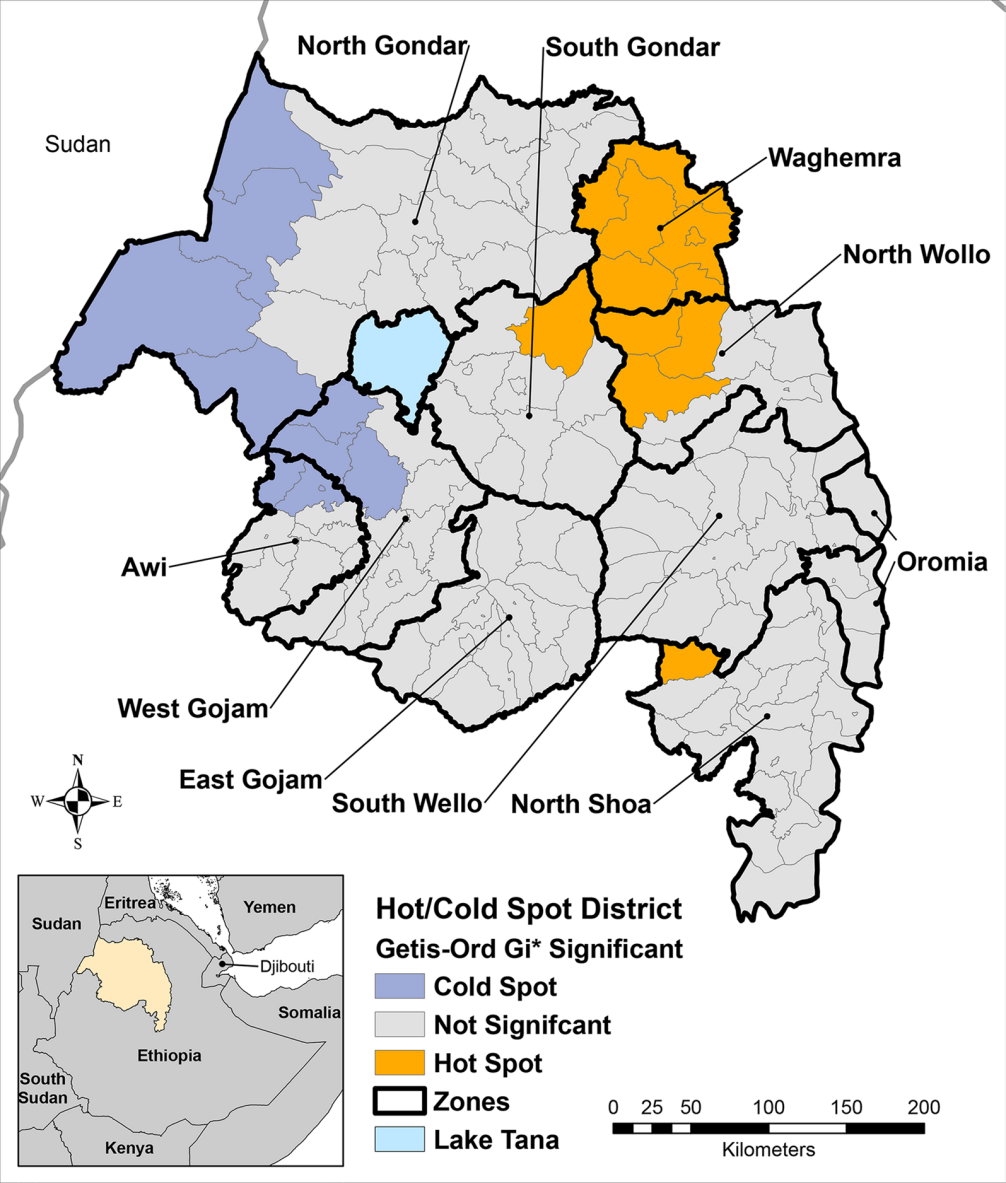
Significant district hot and cold spots of TF prevalence in children aged 1 to 9 years, Amhara, Ethiopia, 2011–2016

**Fig. 2:**
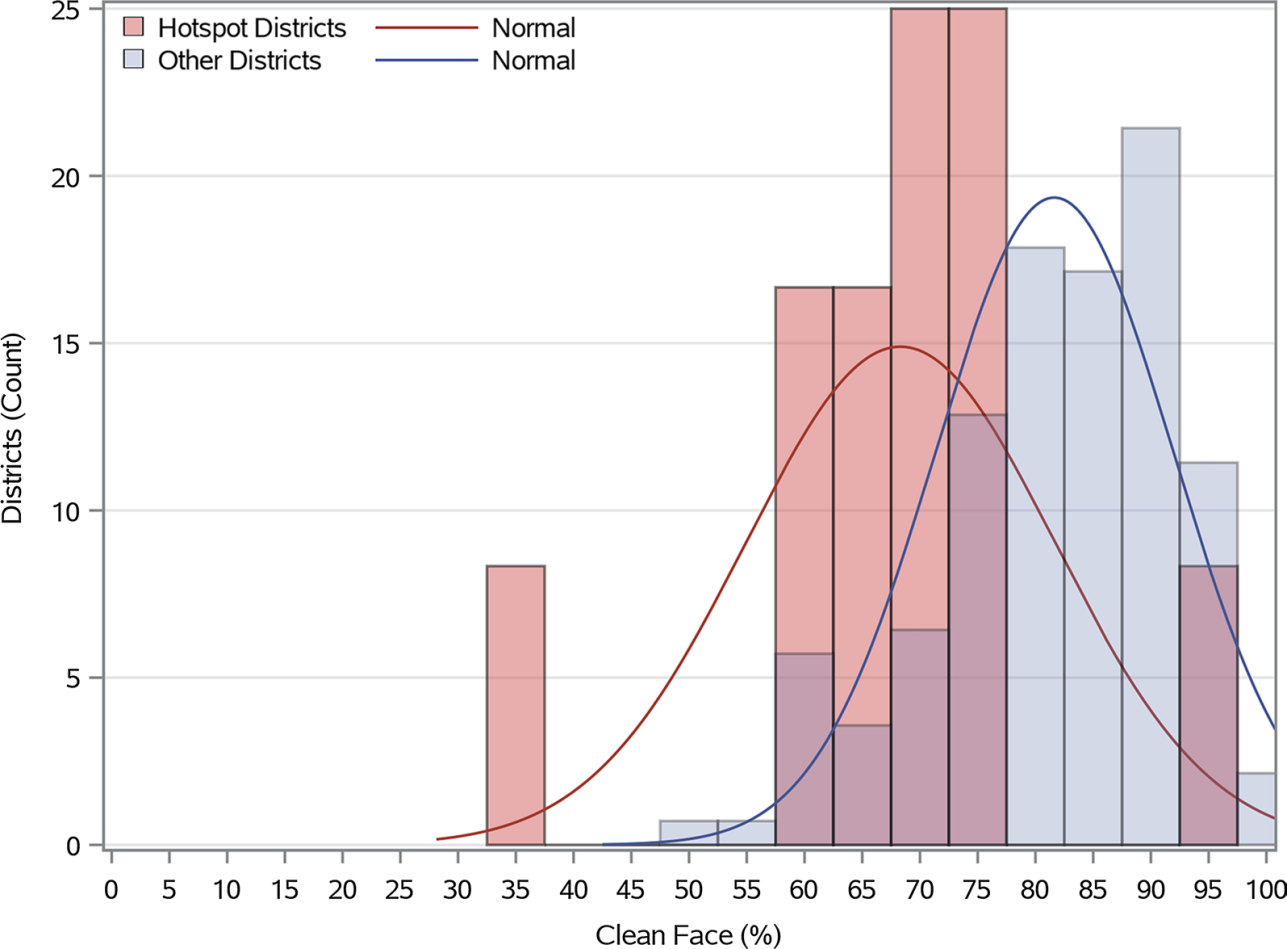
The distribution of the weighted percent of children presenting with clean faces in districts that were statistically significant hotspots compared against all other districts, Amhara, Ethiopia, 2011–2016

Cluster analysis performed using the Getis-Ord G_i_* (d) identified 12 districts (7.9%) as statistically significant hotspots of TF prevalence among children aged 1 to 9 years (Fig. 1). Access to water in less than 30 minutes, access to an improved water source, and mean number of household items owned were negatively associated with district-level hotspots in univariate analysis (Table 1). Furthermore, the percent of villages with a paved road and the average annual precipitation in the districts both had significant negative univariate associations with hotspots. When comparing the distribution of clean face prevalence estimates among districts, the mean clean face prevalence was lower for the TF hotspot districts than for non-hotspot districts (Fig. 2). In the logistic model, clean face was identified to be a statistically significant (odds ratio (OR): 0.91, 95% CI: 0.86–0.96) individual level (negative) predictor. The best-fit district-level multivariate model (model 6, Table 2) included the predictors: clean face (adjusted odds ratio (AOR): 0.90; 95% CI: 0.83–0.97; per 1% prevalence increase), access to water within 30 minutes (AOR: 0.95, 95% CI: 0.91–0.99; per 1% increase), household access to an improved water source (AOR: 0.96; 95% CI: 0.92–, 1.00; per 1% increase) and presence of a health facility in the village (AOR: 0.99; 95% CI: 0.94–1.10; per 1% increase), (AIC = 48.87). This model had good fit (Hosmer-Lemeshow Test = 2.28, *P* = 0.97) and exhibited good discriminatory power from the ROC curve generated for the model, area under the curve, AUC = 0.94.

**Table 1:** Analysis variables weighted to the district and stratified by the outcome of residing in a hotspot *versus* residing in a district that was not a statistically significant hotspot identified using the Getis-Ord Gi*(d) statistic (*n* = 152), Amhara, Ethiopia, 2011–2016

Variable	All districts Mean ± SD	Non-hotspot districts Mean ± SD	Hotspot districts Mean ± SD	Univariate OR (95% CI)
Individual				
Trachomatous inflammation-follicular (%)	25.08 ± 16.19	23.08 ± 14.53	48.54 ± 16.79	1.13 (1.07–1.20)
Children with clean faces (%)	80.58 ± 11.13	81.63 ± 10.31	68.30 ± 13.39	0.91 (0.86–0.96)
Household				
Access to a latrine	52.22 ± 19.95	53.04 ± 20.08	42.60 ± 16.24	0.97 (0.95–1.00)
Access to a water source within 30 minutes (%)	61.58 ± 20.73	63.48 ± 19.52	39.34 ± 22.43	0.95 (0.92–0.98)
Access to an improved water source (%)	55.07 ± 23.35	56.70 ± 22.55	36.10 ± 25.18	0.96 (0.94–0.99)
Crowding (> 6 persons living in the home) (%)	38.22 ± 9.54	37.88 ± 9.49	42.19 ± 9.70	1.05 (0.99–1.12)
Mean number of household items owned	1.48 ± 0.76	1.56 ± 0.74	0.52 ± 0.32	0.01 (0.00–0.11)
Any reported formal education (%)	41.03 ± 16.99	40.73 ± 16.49	44.51 ± 22.69	1.01 (0.98–1.05)
Village				
Health facility (%)	22.53 ± 17.19	24.32 ± 16.24	15.70 ± 23.37	0.97 (0.92–1.01)
Paved road	39.64 ± 24.19	40.78 ± 24.18	20.26 ± 14.99	0.94 (0.89–0.99)
District				
Previous rounds of MDA	6.59 ± 1.64	6.63 ± 1.68	6.25 ± 1.22	0.86 (0.59–1.26)
Climate variables				
Average annual precipitation (mm)	1135.00 ± 278.37	1135.00 ± 278.37	809.50 ± 94.27	0.98 (0.97–0.99)
Average annual temperature (°C)	18.41 ± 3.05	18.41 ± 3.05	19.78 ± 2.25	1.16 (0.97–1.38)
Altitude (m)	2057.91 ± 477.36	2057.91 ± 477.36	1951.42 ± 313.98	1.00 (1.00–1.00)

**Table 2:** Summary of the model selection procedure for finding the best-fit logistic model that predicts membership in a hotspot district using a data driven procedure where the least significant variable was dropped from the model regardless of the hypothesized relationship

Model	Variables analyzed						Model fit	
	Clean	Latrine	H_2_O < 30	iH_2_O	Education	HC Facility	AIC	LRT
1	−X**	+X ns	−X*	−X ns	−X ns	−X ns	51.39	29.94
2	−X**	+X ns	−X**	−X ns	−X ns	–	65.59	30.37
3	−X**	+X ns	−X*	−X ns	–	−X ns	50.66	28.68
4	−X**	+X ns	−X**	−X ns	–	–	63.91	30.06
5	−X**	–	−X*	−X ns	−X ns	−X ns	49.86	29.48
6	−X**	–	−X**	−X ns	–	−X ns	48.87	28.47
7	−X**	–	−X**	−X ns	–	–	63.69	28.27
8	−X**	+X ns	−X**	–	−X ns	−X ns	52.45	26.88
9	−X**	+X ns	−X**	–	–	−X ns	52.84	24.49
10	−X**	–	−X**	–	–	−X ns	50.84	24.49
11	−X**	–	−X**	–	−X ns	−X ns	50.73	26.60
12	−X**	–	−X**	–	–	–	63.62	36.34

*Symbols*: X, variable tested in model; –, variable not included in model; −, negative association; +, positive association; ns, not significant

*Notes*: Variables: Clean (children who presented to examiners without nasal and ocular discharge); Latrine (household has a latrine of any type); H_2_O < 30 (the household has access to a water source within 30 minutes of the home); iH_2_O (the household has access to an improved water source where improved is defined using the sustainable development goals criteria for an improved source); Education (the percentage of households that reported having attended any type of formal education), HC Facility (the presence of any type of health facility within the surveyed village). Model fit: AIC (Akaikeʼs information criterion), LRT (Likelihood ratio test)

*P ≤ 0.05, **P ≤ 0.01

**Fig. 3:**
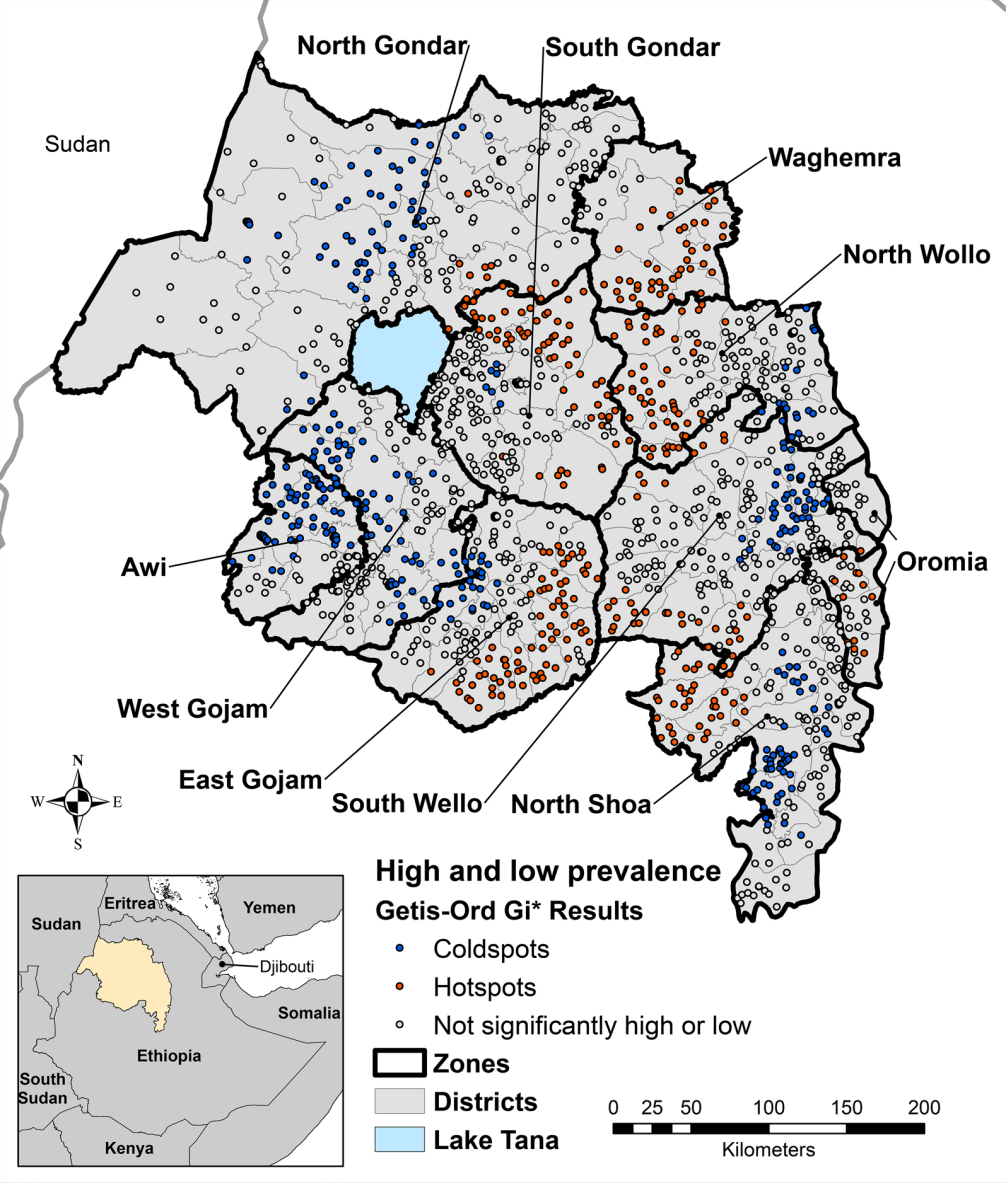
Villages identified as hotspots using the Getis-Ord Gi* (d) statistic and a 25 km neighborhood with 95% Confidence, Amhara, Ethiopia, 2011–2016

Of the 1558 surveyed villages, 325 (20.9%) were identified as having an unusually high prevalence of TF among children aged 1 to 9 years compared to the global average (Fig. 3). The selected multivariate model for explanatory factors in village hotspots (model 20, Table 3) identified clean face, mean count of household items, previous rounds of MDA within the district, average annual precipitation, and average annual temperature as significant explanatory variables. Although not significant, access to water in less than 30 minutes, formal education among household interviewees, and the presence of a paved road were retained in the model as they increased model performance. The model had good fit (Hosmer-Lemeshow Test = 9.68, *P* = 0.29) and exhibited good discriminatory power from the ROC curve generated for the model (AUC = 0.78).

**Table 3:** Multivariate model testing for explanatory factors and the effects on model fit characteristics associated with the residence in a cluster of high TF prevalence villages of Amhara, Ethiopia

Model	Variables analyzed													Model fit	
	Clean	Latrine	H_2_O < 30	iH_2_O	Crowd	Wealth	Education	HC Facility	Paved Road	Prev MDA	Precip	Temp	Altitude	AIC	LRT
1	−X**	+X ns	+X ns	+X ns	−X ns	−X**	−X ns	−X ns	+X ns	+X*	−X**	+X ns	+X ns	1339.76	283.93
2	−X**	+X ns	+X ns	+X ns	−X ns	−X**	−X ns	−X ns	+X ns	+X*	−X**	−X**	–	1338.37	283.33
3	−X**	+X ns	+X ns	+X ns	–	−X**	−X ns	−X ns	+X ns	+X*	−X**	+X ns	+X ns	1339.62	282.07
4	−X**	+X ns	+X ns	+X ns	−X ns	−X **	−X ns	–	+X ns	+X*	X**	+X ns	+X ns	1338.70	282.99
5	−X**	+X ns	+X ns	+X ns	−X ns	−X**	−X ns	−X ns	–	+X *	−X**	+X ns	+X ns	1340.00	281.69
6	−X**	+X ns	+X ns	+X ns	−X ns	−X**	–	−X ns	+X ns	+X*	−X**	+X ns	+X ns	1340.62	281.07
7	−X**	+X ns	+X ns	+X ns	–	−X**	−X ns	−X ns	+X ns	+X**	−X**	−X **	–	1338.17	281.53
8	−X**	+X ns	+X ns	+X ns	−X ns	−X**	−X ns	–	+X ns	+X *	−X**	−X**	–	1337.37	282.32
9	−X**	+X ns	+X ns	+X ns	−X ns	−X**	−X ns	−X ns	–	+X*	−X**	−X**	–	1338.72	280.98
10	−X**	−X ns	+X ns	+X ns	−X ns	−X**	–	−X ns	+X ns	+X**	−X**	−X**	–	1339.27	280.42
11	−X**	+X ns	+X ns	+X ns	–	−X**	−X ns	–	+X ns	+X**	−X**	−X**	–	1337.24	280.46
12	−X**	−X ns	+X ns	+X ns	−X ns	−X**	–	–	+X ns	+X**	−X**	−X**	–	1338.07	279.62
13	−X**	+X ns	+X ns	+X ns	−X ns	−X**	−X ns	–	–	+X*	−X**	−X**	–	1337.57	280.13
14	−X**	−X ns	+X ns	+X ns	–	−X**	–	–	+X ns	+X**	−X**	−X**	–	1337.77	277.92
15	−X**	+X ns	+X ns	+X ns	–	−X**	−X ns	–	–	+X**	−X **	−X **	–	1337.25	278.45
16	−X**	+X ns	+X ns	–	–	−X**	−X ns	–	+X ns	+X**	−X**	−X**	–	1336.60	279.10
17	−X**	+X NS	–	+X ns	–	−X**	−X ns	–	+X ns	+X**	−X**	−X**	–	1338.08	277.61
18	−X**	–	+X ns	+X ns	–	−X**	−X ns	–	+X ns	+X **	−X **	−X **	–	1335.24	280.46
19	−X**	–	–	+X ns	–	−X**	−X ns	–	+X ns	+X**	−X**	−X**	–	1336.11	277.59
20	−X**	–	+X ns	–	–	−X**	−X ns	–	+X ns	+X**	−X**	−X**	–	1334.61	279.08
21	−X**	–	–	–	–	−X**	−X ns	–	+X ns	+X**	−X**	−X**	–	1336.00	275.69

*Symbols*: X, variable tested in model; –, variable not included in model; −, negative association; +, positive association; ns, not significant

*Notes*: Variables: Clean (children who presented to examiners without nasal and ocular discharge); Latrine (household has a latrine of any type); H_2_O < 30 (the household has access to a water source within 30 minutes of the home); iH_2_O (the household has access to an improved water source where improved is defined using the sustainable development goals criteria for an improved source); Crowd (the household has more than 6 residents); Wealth (the mean number of household wealth indicators); Education (the percentage of households that reported having attended any type of formal education); HC Facility (the presence of any type of health facility within the surveyed village); Paved Road (the presence of a paved road within the village); Prev MDA (the number of previous rounds of antibiotic distributed to the district during MDA campaigns); Precip (the average annual precipitation in millimeters for the village); Temp (the average annual temperature in degrees Celsius for the village); altitude (the altitude for the village center). Model fit: AIC (Akaike information criterion), LRT (Likelihood ratio test)

*P ≤ 0.05, **P ≤ 0.01

## Discussion

After SAFE interventions, the distribution of TF among children aged 1 to 9 years in Amhara remained spatially clustered at both the district and the village spatial scales. Hotspots of TF clustering overlapped at both spatial scales in the northeast part of the region. Household access to water was a statistically significant predictor of TF clustering at the district level, and directly observed clean face among children aged 1 to 9 years was a significant predictor at both spatial scales. This suggests that hygiene and water availability could contribute in important ways to the spatial pattern of trachoma throughout Amhara. Along with continuing to provide annual MDA, a continued focus on F and E interventions are likely needed to sustainably eliminate trachoma as a public health problem in the most affected areas of the region.

Starting in 2007, the Trachoma Control Programme in Amhara has scaled up the SAFE strategy to all districts in the region. At scale, the Programme annually delivers approximately 16 million doses of antibiotics, provides health education on face washing and hygiene to approximately 3400 villages and 8000 schools, and assists in the construction of approximately 380,000 latrines [8]. Accordingly, previous reports have demonstrated that increases in household latrines have been observed regionally [8]. However, recently published reports have demonstrated that much of the region still has hyperendemic levels of TF and a considerable burden of infection with *Chlamydia trachomatis,* and thus many more years of SAFE interventions are likely needed [8, 11]. This report better details the pattern and clustering of TF in a post-SAFE setting from a large programme at scale. The identified hotspots may represent areas of the region where the SAFE strategy has yet to be effective. These data will allow the programme to focus on these hotspots to ensure that high MDA coverage is being achieved, and to ensure that F and E efforts are intensified. It is likely that these areas will be the last in the region to eliminate trachoma as a public health problem [13].

The prevalence of children with a clean face was a robust correlate of residence in a trachoma hotspot. Mechanistically, nasal and ocular discharge on children’s faces can serve as a reservoir of infection and can lead to disease transmission [20, 21]. Previous research has shown that poor facial cleanliness including ocular and nasal discharge is consistently associated with both trachoma clinical signs and infection in children [22, 23]. However, of the few published randomized trials focused on facial cleanliness, only one demonstrated a reduction in severe inflammatory trachoma after a face washing intervention [24, 25]. It has been demonstrated over the years that graders in the field can reliably grade clean face, but it has also been shown that clean face does not always predict whether a face has been recently washed [21, 26, 27]. This current study illustrates that once this indicator was aggregated to the more programmatically relevant village or district level in Amhara, clean face was a consistent correlate of TF burden. Although the role of clean face as a trachoma indicator will continue to be debated, transmission reduction through improving hygiene should remain a key component of the SAFE strategy. Promoting knowledge about hygiene behavior is likely not sufficient, rather distinct F and E interventions must be designed with a grounding in behavior change theory, and adapted to each community with local support to create lasting behavioral change [28, 29]. Geographically targeting hotspots would allow programmes to more efficiently deploy these resource intensive projects.

Beyond clean face, several other WASH variables were associated with TF hotspots in these analyses. Increased access to water within 30 minutes was significantly negatively associated with residence in a trachoma hotspot district when controlling for other variables in the model, supporting some prior research [15, 30, 31] but not all [22, 32], that water access is an important predictor of trachoma. The survey question did not enable the respondent to relate whether the water supply was consistently available. Directly observed household water quantity and its allocation for hygiene may be a better health indicator in future studies [33]. Access to an improved water source was not a significant predictor of residence in a hotspot district or hotspot village. It may be possible that the type of water source used for hygiene behaviors is not as important as having access to water, or as important as how that water is used in the household [33, 34]. Household access to a latrine was not a significant predictor of hotspots at the geographical scales considered in our analysis, although previous work has shown links between the presence of sanitation and trachoma [22]. In a previous report from this region, a significantly lower odds of trachoma were found in communities with ≥ 80% community latrine use [35]. These results may have differed from ours due to the different latrine definitions used (presence *vs* use) and the fact that our analysis focused solely on trachoma hotspots. In a 13-country cross-sectional analysis, high community coverage of improved sanitation was associated with lower TF. However, in a sensitivity analysis with any latrine coverage as the exposure, a definition more comparable to ours, a statistically significant association was not observed [36]. The observation of the presence or absence of a latrine without accounting for usage may obscure the exposure/disease relationship and should be further accounted for in future research linking sanitation and trachoma [37, 38].

The quantitative analysis of spatial patterns is a valuable tool to comprehend the complex interrelationships inherent within human populations. Regional differences in psychosocial, contextual, and technological barriers may inhibit habituation of hygiene behaviors and the ability to create a sustained impact on trachoma. The identification of clusters is dependent on the spatial scale analyzed. The differences in the observed percentage of villages compared to the observed percentage of district hotspots could be due to a stronger influence of factors promoting clustering at finer geographical resolution, or the spatial effects might be masked as a result of the modifiable areal unit problem when observing the hotspot results from the district analysis [39]. Surveys were designed to provide district-level estimates, therefore there may have been more variability in the village-level analysis which may have led to different results at different spatial scales. We observed, for example both hotspot and non-hotspot villages within the same district. Although impact surveys are designed to account for this village-level variability, it is possible that high-prevalence subpopulations could be missed by district-level surveys causing problems for control programmes. Interpreting individual village data from district-level surveys should be done with caution however, as it is expected that even districts reaching elimination may contain villages with elevated levels of *C. trachomatis* infection as part of a naturally occurring distribution [40].

This study used cross-sectional data and therefore causal links between SAFE interventions and trachoma prevalence could not be determined. Since pre-SAFE baseline surveys were not conducted at the district level, it was not possible to conduct longitudinal analyses. Future studies using repeat impact survey data will be needed to better understand the contributions of these interventions. All multivariate models at the village-level showed that more rounds of MDA were associated with higher likelihood of a village being a TF hotspot. Many of those hotspot villages were located in districts which were among the first enrolled in the SAFE programme, and which had high levels of TF initially [7]. The high trachoma prevalence in these districts would have warranted more MDA rounds as per the WHO guidelines and thus this result may have been due to reverse causality. This analysis also depended on the outcome TF, a clinical sign which has been shown to not correspond well to *C. trachomatis* infection in a post-SAFE setting [41]. Despite this, study teams did include certified trachoma graders who underwent the same training and were required to pass a field reliability exam before each survey round. Lastly, the examination of relationships between WASH variables and TF was a secondary aim of these surveys, and the large size of the dataset may have allowed for detecting statistically significant relationships despite small effect sizes. Although cross-sectional modeling such as this is a common practice in the trachoma literature, there are more robust ways to test these relationships such as a WASH trial that is currently underway in some of the most challenging districts of the region [42].

## Conclusions

The Trachoma Programme in Amhara has made progress by reducing the prevalence of the disease in many areas since fully implementing the SAFE strategy; however, the distribution of trachoma was not spatially random. This study used data from 69,236 children aged 1 to 9 years across the entire region of Amhara to evaluate spatial relationships at a large-scale, between districts, and then further compared the results to a finer-scale, village-level analysis. Important WASH variables were correlates of trachoma hotspots in this region including facial cleanliness and water access. Spatial analysis is a powerful tool to identify geographical areas in greatest need of intervention and can help to reduce operational costs by targeting the most appropriate interventions locally. Directing F and E interventions to areas with the most severe trachoma will likely accelerate the elimination of trachoma as a public health problem.

## Data Availability

The complete dataset analyzed in this paper is not publicly available in its entirety due to ongoing analyses by other authors covering separate but related topics. Datasets required to reproduce analyses and results presented in this study are available from the corresponding author upon reasonable request.

## Abbreviations

AIC: Akaike information criterion
AOR: adjusted odds ratio
AUC: area under the curve
CI: confidence interval
GET2020: Global Alliance to Eliminate Blinding Trachoma by 2020
GPS: global positioning system
IRB: internal review board
OR: odds ratio
MDA: mass drug administration
ROC: receiver operating characteristics
SAFE: surgery, antibiotics, facial cleanliness, and environmental improvement
TF: trachomatous inflammation-follicular
WASH: water sanitation and hygiene
WHO: World Health Organization
